# Pro-metastatic functions of lipoproteins and extracellular vesicles in the acidic tumor microenvironment

**DOI:** 10.1007/s10555-019-09786-5

**Published:** 2019-02-15

**Authors:** Anna Bång-Rudenstam, Myriam Cerezo-Magaña, Mattias Belting

**Affiliations:** Department of Clinical Sciences Lund, Section of Oncology and Pathology, Lund University, Barngatan 4, SE-221 85 Lund, Sweden

**Keywords:** Cancer, Acidosis, Metastasis, Proteoglycans, EVs, Lipid metabolism

## Abstract

Although the overall mortality in cancer is steadily decreasing, major groups of patients still respond poorly to available treatments. The key clinical challenge discussed here relates to the inherent capacity of cancer cells to metabolically adapt to hypoxic and acidic stress, resulting in treatment resistance and a pro-metastatic behavior. Hence, a detailed understanding of stress adaptive responses is critical for the design of more rational therapeutic strategies for cancer. We will focus on the emerging role of extracellular vesicles (EVs) and lipoprotein particles in cancer cell metabolic stress adaptation and how these pathways may constitute potential Achilles’ heels of the cancer cell machinery and alternative treatment targets of metastasis. In this context, common extracellular lipid uptake mechanisms, involving specific cell-surface receptors and endocytic pathways, may operate during remodeling of acidic atherosclerotic plaques as well as the tumor microenvironment. The role of endocytosis in regulating the cellular response to hypoxic and acidic stress through spatial coordination of receptor proteins may be exploited for therapeutic purposes. As a consequence, molecular mechanisms of endocytosis have attracted increasing attention as potential targets for tumor specific delivery of therapeutic substances, such as antibody–drug conjugates. The identification of internalizing surface proteins specific to the acidic tumor niche remains an unmet need of high clinical relevance. Among the currently explored, acidosis-related, internalizing target proteins, we will focus on the cell-surface proteoglycan carbonic anhydrase 9.

## The acidic tumor microenvironment

**Fig. 1:**
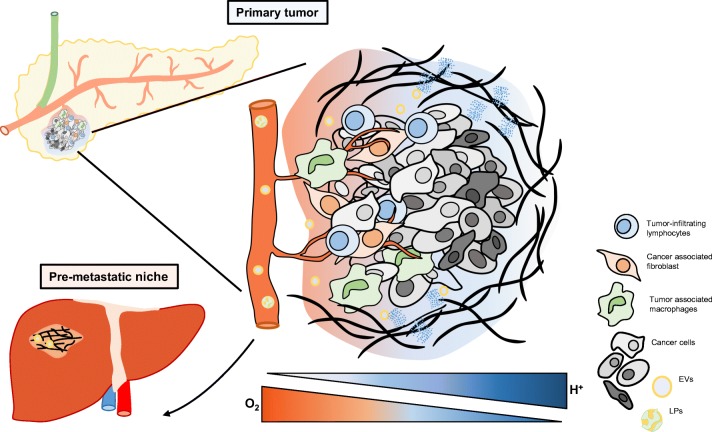
Schematic overview of the tumor microenvironment. Cancer cells and their stroma together shape a complex environment, a process that is largely facilitated by adaptive responses to hypoxia and acidosis. A close interplay between stress-adapted cancer cells, ECM components, and stromal cells drives disease progression by tissue remodeling in the primary tumor as well as in distant tissues that form the pre-metastatic niche, here exemplified by pancreatic cancer dissemination to the liver. ECM extracellular matrix, EVs extracellular vesicles, LPs lipoproteins

Cancer develops in a complex milieu, also known as the tumor microenvironment (TME), where the malignant cells and its stroma, including endothelial cells, pericytes, fibroblasts, immune cells, and the extracellular matrix (ECM), coexist during tumor evolution (for review, see [1]). Oncogenic events initially drive malignant development. However, stress factors such as hypoxia and acidosis have a key role early on in tumor development that, together with genetic factors, further fosters the selection of pro-metastatic subpopulations of highly heterogeneous tumors (Fig. 1). We will give a brief overview of the local metabolic functions of tumor hypoxia and extracellular acidosis and their role in ECM remodeling. A more detailed discussion will then focus on different aspects of tumor acidosis and lipid metabolism, and acidosis as a modulator of several functional aspects of malignant features involving EVs and lipoproteins, both locally in the primary tumor and at more distant locations, including the pre-metastatic niche (PMN). Finally, we will discuss potential therapeutic implications connected with tumor adaptive responses to hypoxia and acidosis.

## Structural remodeling of the acidic tumor niche drives metastasis

**Fig. 2:**
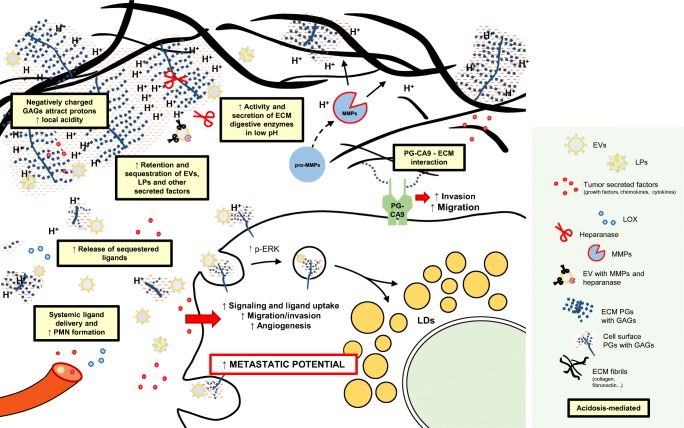
Extracellular acidosis regulates cancer cell–extracellular ligand interactions, enhancing pro-metastatic behavior. The invasion of surrounding tissue by tumor cells relies on ECM digestion. TME acidosis induces protease and heparanase secretion and activity, both as soluble proteins and bound to EVs. Acidosis also enhances the sequestration of pro-tumorigenic ligands in the ECM, including growth factors, cytokines, and chemokines, as well as EVs and LP particles. Enhanced ligand release ultimately leads to increased local availability and induction of receptor signaling and ligand internalization, contributing to cancer cells stress adaptation. Further, released, pro-tumorigenic ligands will enter the circulation and arrive at distant sites to induce PMN formation. PG-CA9 is induced at the cell surface of acidic cells and not only is a major player in pH homeostasis but also takes part in cell–ECM interactions to promote cancer cell invasion and migration. Acidosis-mediated effects are represented with yellow boxes. ECM extracellular matrix, EVs extracellular vesicles, GAG glycosaminoglycan, LDs lipid droplets, LOX lysyl oxidase, LPs lipoproteins, MMPs matrix metalloproteinases, PGs proteoglycans, PG-CA9 proteoglycan version of carbonic anhydrase 9, PMN pre-metastatic niche, TME tumor microenvironment

As a key component of the TME, the ECM comprises the three-dimensional network of extracellular molecules that provide structural and biochemical support for surrounding cells. During tumor evolution, both the content and the organization of the ECM are altered in close concert with the hypoxic and acidic tumor landscape [2–4], ultimately contributing to metastasis and disease progression (Fig. 2). The major components of the ECM include fibrous proteins such as collagens, elastin, fibronectin and laminin, and proteoglycans (PGs) [5]. The interplay between the ECM and the acidic TME is considered important in several aspects of metastasis; detachment from the surrounding stroma, migration, and local invasion are some of the first steps a cancer cell must take to further disseminate to and finally thrive in a new tissue compartment. Many of the early metastatic events are supported by the “imbalance” in intracellular and local extracellular pH, a phenomenon referred to as the “reversed pH gradient.” This is characterized by a low extracellular pH (pHe) concomitantly with a slightly alkaline intracellular pH (pHi) (further reviewed in [3, 6]). An acidic extracellular pH stimulates processes important for cancer cell migration by the induction and activation of ECM digestive enzymes, including matrix metalloproteinases (MMPs) and cathepsins, i.e., acidosis-activated proteases. Under physiological conditions, MMPs function to control homeostasis of connective tissue in various organs [7]. In the acidic TME, however, the abundance and activity of these enzymes are highly induced to serve as key players in the remodeling of the ECM [8–11]. The degradation of the ECM is further accelerated by the activity of heparanase, i.e., an acidosis-activated endoglycosidase that degrades heparan sulfate (HS) glycosaminoglycans (GAGs) [12]. During matrix degradation, sequestered pro-tumorigenic factors, including growth factors, cytokines, and chemokines, are liberated that further enhance pro-invasive signaling of tumor cells [13, 14]. Notably, non-classical signaling molecules of the TME, including EVs and lipoproteins, are known to interact with PGs in the ECM. As a consequence, low pH conditions, favoring increased protease and heparanase activity in the TME, should also influence the availability of these nanoparticle structures with potential signaling and metabolic functions, locally as well as systemically (further discussed below).

## Metabolic rewiring is associated with acidification of the tumor niche

**Fig. 3:**
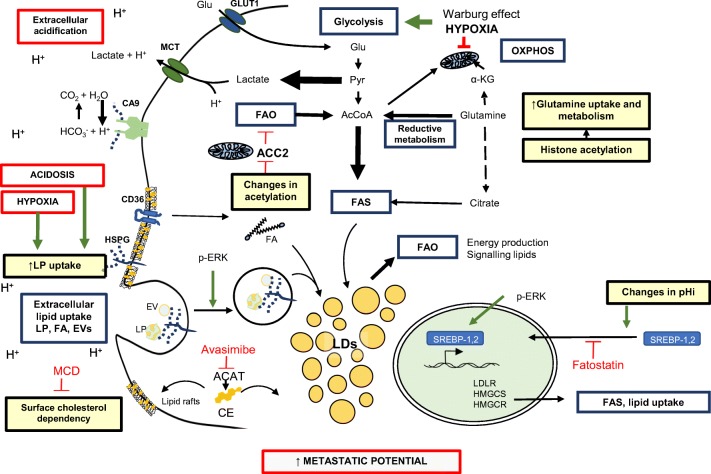
The tumor microenvironment influences the metabolic status of cancer cells with consequences for their metastatic potential. Both exogenous lipids (e.g., FAs, LPs, or EVs) and endogenous lipids (obtained by FAS) can be stored in LDs, conferring a lipid-loaded cancer cell phenotype, which correlates with an increased metastatic potential. TME conditions can potentiate different aspects of lipid metabolism. Acidosis and hypoxia stimulate exogenous LP internalization *via* HSPGs, in a process that involves p-ERK signaling. The SREBP-dependent pathway represents a main lipogenic program and has been linked to metastasis. SREBP can be activated under acidic conditions through changes in pHi. Changes in ACC2 acetylation allow FAO to occur concomitantly with FAS in acidosis-adapted cells. Further, increased glutamine metabolism in acidosis-adapted cells due to changes in histone acetylation serves as a source of AcCoA that fuels FAS. Drugs targeting different lipid pathways constitute interesting therapeutics targeted at metastatic cells (shown in red). Extracellular acidosis-mediated effects are represented with yellow boxes. α-KG alpha-ketoglutarate, ACC2 acetyl-CoA carboxylase, ACAT Acyl-CoA cholesterol acyltransferase, AcCoA Acetyl CoA, CA9 carbonic anhydrase 9, CE cholesteryl ester, EV extracellular vesicle, FA fatty acid, FAO fatty acid oxidation, FAS fatty acid synthase, Glu glucose, GLUT1 glucose transporter 1, HMGCR HMGCoA reductase, HMGCS HMGCoA synthase, LDLR low-density lipoprotein receptor, LDs lipid droplets, LP lipoprotein, MCD methyl-β-cyclodextrin, MCT monocarboxylate transporter, OXPHOS oxidative phosphorylation, pHi intracellular pH, Pyr pyruvate, SREBP sterol regulatory element-binding protein

Changes in cell metabolism are a hallmark of cancer [15] as tumor cells rely on their ability to rewire their anabolic and catabolic pathways in order to obtain both energy in the form of ATP and complex molecules like lipids for processes such as cell division (Fig. 3). A well-established metabolic feature of tumor cells is their preferential use of glycolysis over oxidative phosphorylation (OXPHOS) for ATP production. In the core of solid tumors, hypoxic cells rely on glycolysis because of their inability to have a functional OXPHOS due to oxygen unavailability. Even under normal oxygen conditions, cancer cells rewire their metabolism to “aerobic glycolysis” (Warburg effect). Some tumor types, however, still rely on mitochondrial respiration as a major source of ATP [16, 17]. The increased glucose metabolism in solid tumors for energy production translates into lactate production from pyruvate fermentation and constitutes the driving force of extracellular acidification. CO_2_ released by cancer cells undergoing mitochondrial respiration further contributes to acidosis by its hydration into HCO_3_^−^ + H^+^, catalyzed by carbonic anhydrases [18].

The immense lactate production that occurs in glycolytic, hypoxic areas has been studied as a nutrient source in solid tumors. Lactate can be taken up by cancer cells through monocarboxylate transporters (MCTs) and be utilized for energy production through oxidative metabolism. Interestingly, a symbiotic relation has been postulated between glycolytic, lactate-producing cancer cells, and cells relying on oxidative metabolism in areas where O_2_ is available. “Oxidative cells” may internalize lactate through MCT1 in favor of glucose and utilize it for mitochondrial oxidation. In this way, glucose availability is increased for the glycolytic, hypoxic cells. Targeting lactate metabolism in the oxygenated areas by MCT1 blockade increases glucose in these cells and indirectly causes hypoxic cell death due to decreased remnant glucose availability [19].

In the acidic TME, increased free fatty acid uptake in the form of palmitate was reported, and acidosis-adapted cells use palmitate as a metabolic substrate for mitochondrial respiration [20]. In the same study, Corbet et al. suggest that fatty acid oxidation (FAO) occurs concomitantly with FA synthesis in acidosis-adapted cells, which in healthy tissues are usually mutually exclusive. Changes in the protein acetylome of acidosis-adapted cells may downregulate acetyl CoA carboxylase (ACC2) that would normally prevent FAO of newly synthesized lipids [20]. In this scenario, FAO is the major source of acetyl CoA (AcCoA) for the mitochondria, which in the presence of oxygen is metabolized by OXPHOS. Moreover, increased glutamine uptake, together with a positive regulation of glutamine metabolism enzymes, was suggested in acidosis-adapted cells, and this shift to reductive glutamine metabolism was connected with a change in histone acetylation of hypoxia-inducible factor (HIF)-responsive genes [21]. Notably, the increased AcCoA production by reductive glutamine metabolism from α-ketoglutarate constitutes the substrate for *de novo* lipogenesis and fuels this pathway under acidic conditions, as it has been shown previously in hypoxic stress [22]. Acidosis-adapted cells are shown in this context to be “mitochondrially active” through TCA cycle utilization of AcCoA from FAO and glutamine reductive metabolism. Under these conditions, mitochondria-inhibiting agents, like metformin, would be interesting candidates as repurposing drugs against the well-oxygenated acidic tumor niche, however, with less activity in the lactate-driven acidic tumor core [16].

## Lipids as fuel for metastasis: role of tumor acidosis

Building on the findings on glucose metabolism, lipid metabolism has gained increasing interest in cancer due to numerous studies that link changes in tumor cell lipid availability to the metastatic potential of malignant cells. Lipids can accumulate in the cytoplasmic compartment in organelles referred to as “lipid droplets” (LDs), composed of neutral lipids and cholesteryl esters, and surrounded by perilipins and other specific coat proteins. Aberrant LD accumulation has been shown in a variety of cancer types [23–26]. Importantly, LD accumulation is affected by environmental stress like acidosis and hypoxia [27, 28] as well as chemoresistance [26] and can be mediated both by *de novo* lipogenesis and extracellular lipid uptake [27, 29]. Sterol regulatory element-binding proteins (SREBPs) are master regulators of a lipogenic program that controls cholesterol homeostasis and regulates the expression of target genes for lipid uptake (e.g., LDLR) and cholesterol biosynthesis (HMGCoA synthase and reductase). Extracellular acidosis has been suggested to indirectly activate the SREBP pathway through SREBP-2 nuclear translocation. Despite other studies describing an increased pHi concomitant with extracellular acidosis [21, 30], studies of acidosis-adapted cancer cells from different tumor origins suggest that a decrease in pHi results in SREBP-2 related gene expression activation. However, the mechanism linking changes in pHi and SREBP activation are still elusive [31]. Other studies revealed that increased expression of lysophosphatidylcholine acyltransferase 2 (LPCAT2), i.e., a key enzyme in phosphatidylcholine synthesis, correlated with an increased LD content and was shown to co-localize with LDs in colorectal cancer. Interestingly, the LD phenotype may promote chemoresistance by a mechanism involving ER stress inhibition, and inhibition of LD formation reversed the resistance phenotype [26]. LD-loaded cancer cells have shown increased metastatic potential in an experimental lung cancer model [27], and excessive LD accumulation was found in patient prostate cancer metastases [32]. Moreover, inhibition of cholesterol esterification significantly suppressed the development of metastatic lesions in experimental prostate cancer models, possibly through downregulation of the Wnt/β-catenin pathway. PTEN is an established regulator of the SREBP lipogenic pathway and is also commonly deleted in prostate cancer [23]. In a model of metastatic prostate cancer, the common co-deletion of *PML* and *PTEN* was suggested to drive metastatic potential through SREBP program activation. The potential of SREBP as a therapeutic target was suggested from treatment studies with fatostatin, which inhibits SREBP-dependent *de novo* lipogenesis. SREBP inhibition resulted in reduced prostate cancer cell growth and metastasis [23]. In another study on metastatic pancreatic cancer [29], the aberrant cholesteryl ester accumulation in LDs was targeted with avasimibe, which prevents free cholesterol esterification by inhibiting acyl-CoA cholesterol acyltransferase-1 (ACAT-1). Migration and invasion of pancreatic cancer cells were significantly reduced by avasimibe, and metastatic dissemination to the lymph nodes and liver was significantly reduced. This was explained by free cholesterol accumulation and elevated ER stress, further leading to apoptosis [29, 33]. ACAT-1 inhibition has also shown promising results in a glioma model, as free cholesterol accumulation after avasimibe treatment resulted in increased tumor cell death and prolonged animal survival [34]. In contrast, studies with triple negative breast cancer cells showed that LD consumption through FAO and CUB-domain containing protein 1 (CDCP1) resulted in decreased lipid accumulation and increased metastatic potential [35]. Although the exact role of LD consumption in the metastatic process remains to be elucidated, LDs may serve as a substrate reservoir of signaling lipid metabolites. As extensively described [36], signaling lipids like eicosanoids, including prostanoids and leukotrienes, have been linked to different steps of metastasis development [36]. The increased abundance of lipid substrates for their synthesis may increase their potential as signaling entities in tumor development. Further studies will be required to unravel how acquisition *vs.* consumption of LDs in the acidic TME may be involved in promoting the metastatic potential and how this differs depending on tumor type.

The above studies mostly concern the role of endogenous lipid metabolism. However, several studies point at the role of exogenous lipid sources in metastasis. CD36, i.e., a hypoxia induced scavenger receptor for extracellular lipids [37–39], takes part in fatty acid and cholesterol uptake as well as in signaling transduction of FA metabolism. In a study by Pascual et al., slow cycling cells, with the greatest tumor initiating potential, overexpressed a repertoire of lipid metabolism genes, including CD36. CD36 targeting with neutralizing antibodies resulted in metastasis remission [40]. These effects could be attributed to the lipotoxicity observed around LD-laden cells in the metastatic niche. It was hypothesized that CD36 is necessary for the initial steps of metastasis and that CD36 blockade leads to LD accumulation and lipotoxicity. Ladanyi et al. [41] reported that resident adipocytes of the peritoneal cavity induced CD36 expression in ovarian cancer cells, which associated with increased fatty acid uptake and accumulation. Accordingly, silencing of CD36 resulted in impaired invasion, migration, and colony forming capacity [41]. Other studies have implicated a role for fatty acid binding protein 4 (FABP4), i.e., another hypoxia-induced lipid transport protein [42], in ovarian cancer metastasis. Overexpression of FABP4 in metastatic cells allowed efficient internalization of fatty acids from adipocytes of the metastatic niche, which was associated with enhanced cell growth [43].

## Proteoglycans are major binding partners of pH-sensitive ligands

PGs are present in virtually all mammalian tissue compartments and constitute a large family of proteins characterized by the covalent conjugation of one or several sulfated GAG polysaccharide chains. GAGs are composed of repeating disaccharide units of amino sugar (GlcNAc in heparan sulfate or GalNAc in chondroitin sulfate) linked to uronic acid (GlcA or IdoA). Sulfate groups are attached at discrete positions along the GAG polysaccharide chain that together with carboxyl groups yield a highly polyanionic structure prone to interactions with sequences or patches of basic amino acids present in a wide variety of protein ligands [44]. Being present intracellularly, at the cell membrane and in the ECM, PGs participate in finely tuned electrostatic interactions. In cancer, the conformations of these structures may be highly modulated, altering structural as well as functional properties involved in tumor development and progression [14, 45, 46]. Although it is unlikely that the negative charge of the sulfate and carboxyl groups (p*K*a < 2.0 and 3.0–4.4, respectively [47]) of PGs would be affected by pH conditions of the TME (which in more extreme cases may reach below pH 6 [48]), one could still argue that the negatively charged GAG chains may influence the local distribution of free protons in the TME [49]. Thus, by reducing the diffusion capacity of positively charged ions in the ECM, PGs could contribute to the extracellular acidification in their direct vicinity [50]. More importantly, increased protonation of histidine residues of protein ligands of the acidic TME would directly favor PG binding with consequences for their diffusion capacity in the ECM and internalization through PG-mediated endocytosis. We will next introduce PG-binding lipid particles that emerge as novel key players in TME remodeling and metastasis.

## EVs and lipoproteins—PG binding nutrients and signalosomes of the TME

EVs are phospholipid-bilayer particles produced and found in most cells and tissues (see also Logozzi et al., this volume). These nanoparticles have a well-established role in intercellular communication, carrying nucleic acids, proteins, and lipids to neighboring as well as distant cells, thereby influencing a multitude of biological processes in both normal physiology and different pathological settings. EVs are usually classified according to their biogenesis, size, and method of release, where exosomes (30–150 nm in diameter) are, by definition, generated intracellularly within multivesicular bodies and are to be distinguished from microvesicles (MVs) (generally 100–1000 nm in diameter), which are shed from the plasma membrane, and from apoptotic bodies released from dying cells. However, the exact distinction between exosomes and MVs is still incomplete, partly due to inconsistent methods of purification, as well as incomplete understanding of their biogenesis, and the two terms are sometimes used interchangeably [51]. Here, we will use the common term EVs for both particles, unless otherwise stated.

The main classes of lipoproteins, i.e., HDL, LDL, VLDL, and chylomicrons, are in contrast to EVs surrounded by a single phospholipid membrane. These particles are classically regarded as carriers of lipids to peripheral tissues. LDL as well as other lipoproteins have been shown to interact with PGs, more specifically cell-surface heparan sulfate proteoglycans (HSPGs), for further clearance through endocytosis. Although the precise contribution of HSPGs in the uptake of lipoproteins is still under investigation, it is known that HSPGs are required for efficient lipoprotein internalization, either as independent receptors or in concert with classical cell receptors, e.g., LDLR, LPR1 VLDLR, SRB1, and CD36 [52–54]. Notably, EVs and lipoproteins, mainly LDL, have several similar features (for review, see ref. [55]); apart from similarities in size and density, increasing evidence shows that these endogenous nanoparticles play key roles in cancer cell adaptation to acidosis and hypoxia (see below). Consistent with this idea, we and others have found that cancer cell-derived EVs, more specifically exosomes, express HS-binding proteins [56], bind to heparin (a highly sulfated HSPG mimetic), and are dependent on HSPGs for their efficient uptake [57]. From previous studies, a central role of PGs, more specifically HSPGs, as key players has emerged not only in the direct interaction but also in facilitating internalization and intracellular trafficking of endogenous nanoparticles of the TME [58–60]. How alterations in the structure and distribution of PGs in the acidic tumor niche regulate these functions is an interesting area for future studies.

## What can we learn from atherosclerosis?

Although the detailed uptake mechanisms as well as the general impact of extracellular proton concentration on EV-to-cell and LDL-to-cell functions remain to be elucidated, some clues may be offered from another pathological process distinguished by anaerobic metabolism and acidosis: the atherosclerotic plaque. Malignant tumors and atherosclerotic lesions are different in many aspects, but there are some striking analogies between their pathological evolutionary trajectories [61, 62]. In addition to a hypoxic/acidic microenvironment, chronic inflammation and intracellular LD accumulation [63, 64] are common features. In atherosclerosis, the importance of lipid abundance in disease initiation and progression, mainly involving LDL, is well established, and EVs have attracted increasing interest as potential players in disease progression [65]. Several studies of atherosclerosis have shown that the affinity of LDL for PGs is quite low at neutral pH and is significantly enhanced with increasing extracellular acidity [66, 67]. Interestingly, LDL clearance by macrophages and smooth muscle cells, ultimately leading to foam cell formation, has been shown to be enhanced in both acidic and hypoxic atherosclerotic conditions, mainly through particle as well as HSPG receptor modifications [39, 68, 69]. In the tumor setting, we recently reported a role of HSPGs in the adaptive response of tumor cells to extracellular hypoxia and acidosis through increased internalization of lipoproteins, resulting in a LD storing phenotype that was associated with augmented spheroid formation and metastatic capacity [27]. Acidic extracellular pH could enhance the local availability of EVs/lipoproteins by increasing ligand retention as well as by directly affecting the PG-to-ligand electrostatic interaction, inducing binding, internalization, as well as affecting downstream signaling events. Although low pH is not considered to alter the PG net negative charge as such, enhanced proton accumulation in the vicinity of PGs (see above) could alter the net positive charge of surrounding ligands, a scenario that already has been discussed in the context of atherosclerosis [69]. Generally, sequences of positively charged arginine and lysine residues of ApoB-100 are known to be responsible for lipoprotein–PG interaction [70, 71]. However, protonation of histidine side chains (p*K*a of approximately 6.0) in ApoB-100 may, at least partly, explain the observed increase in lipoprotein–PG binding in the acidic atherosclerotic microenvironment [69]. Notably, EVs have been shown to be enriched in specific lipoprotein ligands, including ApoE [72], known to bind HSPGs [73, 74], and net charge alterations of ApoE by acidic pH can be expected. Also, heat shock protein 90 (HSP90), i.e., another EV-enriched protein, was found to be enhanced in cancer cells cultured at low pH conditions with consequences for cancer stem cell malignancy [75]. Indeed, HSP90 has been shown to interact with cell-surface HSPGs [76], and HSP90-containing EVs were isolated from tumor cells [77], especially when derived from low-pH cell cultures [78]. This motivates further studies on the role of HSP90 in EV intercellular communication in the acidic tumor niche.

## Increased release of tumor promoting EVs in the stressed TME

An increased release of EVs in response to hypoxia was reported [79–81], and more recently, the role of extracellular acidity in EV release has been explored. In several melanoma models, decreased extracellular pH was shown to induce EV secretion, and this was dependent on the metastatic capacity of donor cells [78, 82]. More recently, the influence of an acidic microenvironment on EV release was comprehensively evaluated, including several human tumor cell lines from colon, breast, and prostate cancers as well as melanoma and osteosarcoma. This study found an increase in EV release at acidic (pH 6.5) culture conditions as compared with physiological pH conditions, independently of tumor type [83]. Taken together, these studies point towards a general induction of EV secretion under acidic as well as hypoxic TME stress, which is in line with the idea that EV secretion is associated with the degree of tumor malignancy [84]. It has been speculated that enhanced EV secretion under acidic stress could work in favor of cell survival through elimination of toxic substances that otherwise would accumulate in stressed cells [85]. Apart from an induced EV secretion in acidic conditions, low pH was shown to increase EV protein and RNA stability [86], suggesting a more potent transfer of signaling molecules by acidosis-derived EVs.

## Changes in the internalization mechanisms of acidic cancer cells: implications for EV uptake

As previously discussed, the importance of enhanced exploitation of exogenous nutrient sources, including lipoproteins and EVs, as well as other macromolecules in malignant tumors is gaining increased interest [87, 88]. Specifically, the role of macropinocytosis has been elaborated in cancer cells harboring oncogenic *RAS* mutations where extracellular protein is internalized through macropinocytosis and degraded to provide nutrient supply and cellular growth [89, 90]. The function of macropinocytosis as a feeding mechanism of extracellular macromolecules provides a new perspective in cancer cell metabolic reprogramming. Few studies have explored how TME features, including hypoxia and acidosis, may regulate the uptake of EVs, lipoproteins and other macromolecules that may occur *via* multiple processes including phagocytosis, macropinocytosis, receptor-mediated endocytosis, and direct membrane fusion [91, 92]. We have provided evidence that EVs mainly enter cells *via* lipid raft-mediated endocytosis through signaling activation of a MAPK-dependent route, and that this pathway is negatively regulated by caveolin-1 [93]. Further, we reported the importance of HSPGs as major internalizing receptors involved in EV uptake and, more importantly, in hypoxia/acidosis-induced LDL uptake, where signaling activation of the MAPK pathway modulated ligand endocytosis [27, 57]. Stress induced LDL uptake was further shown to be linked to an increased metastatic potential [27] and together with the findings on MAPK involvement in lipogenesis [23], these studies strengthen the role of MAPK in lipid accumulation and metastatic potential of cancer cells.

So, how could the lipid raft-mediated endocytosis be modulated by acidosis? The levels of cholesterol in cancer cells are essential not only as building blocks for membrane formation during cell division and as substrates for signaling molecules but also as key constituents of lipid raft membrane domains [94]. Extracellular acidosis can modulate membrane lipid distribution as shown by an increased cholesterol abundance in the plasma membrane of acidosis-adapted glioblastoma cells, with potential consequences for lipid raft structure organization and cell signaling. Tumor cell survival was dramatically reduced after cholesterol depletion by methyl-β-cyclodextrin (MCD) treatment, suggesting an increased dependence on membrane cholesterol in acidosis-adapted cells [95]. Similarly, the integrity of lipid raft domains is important for epithelial-to-mesenchymal transition (EMT) regulation, and lipid raft disruption could lead to decreased invasiveness and chemoresistance in pancreatic tumor initiating cells [96]. Interestingly, in human glioblastoma tumor samples, an association was suggested between HMG-CoA-reductase (HMGCR), the rate-limiting enzyme in cholesterol biosynthesis, and LAMP2, a suggested marker for acidosis [30], further supporting a dependency of acidic cancer cells on cholesterol levels and that this pathway is a relevant therapeutic target of stressed tumor niche [95].

The lipid membrane organization of EVs may be similarly affected by alterations in extracellular pH, ultimately influencing EV delivery to recipient cells in the acidic TME. Indeed, an increased EV uptake through membrane fusion has been suggested in acidic conditions [82]. Although EV-cell fusion generally is considered to be limited due to high rigidity of the EV membrane, altered EV lipid organization in low pH conditions could affect EV-cell fusion potential. A study examining EV membrane fluidity by fluorescence anisotropy in different pH conditions found reduced membrane rigidity at lower pH conditions [97]. As fusion between membranes is facilitated by similar membrane fluidity, decreased pH could be suspected to promote EV delivery. Notably, treatment with the lipid metabolism inhibitor fatostatin was shown to decrease EV production [98], supporting an interplay between donor cell lipid metabolism and EV-dependent intercellular communication [99]. Further, macropinocytosis and phagocytosis are also regulated by extracellular pH conditions [100, 101]. In macrophages, an increased phosphatidylserine-dependent phagocytic capacity, dependent on acidosis-induced stabilin-1, was reported [102]. However, additional studies will be required to provide more firm conclusions regarding how these pathways are regulated in the acidic tumor niche with associated effects on EV transfer.

## EVs promote hypoxia and acidosis-mediated tumor development and metastasis

Several studies have started to unravel potential functions of EVs in the acidic tumor niche, both locally and at distant sites in the PMN. The metabolite composition of EVs was shown to modulate the metabolism and serve as an energy source in recipient cells [103], e.g., hypoxia-derived EVs showed an enhanced triglyceride level [98]. Further, EVs from hypoxic adipocytes showed an increased content of lipogenic enzymes, and this metabolic phenotype was transferred to recipient cells [104]. It may be concluded that EVs have the potential to favor a lipid loaded phenotype not only through the transfer of their lipid content but also through pleiotropic effects imposed by the transfer of metabolic substrates and enzymes into recipient cells. Peppicielli et al. suggested that conditioned media from acidic tumor cells can stimulate invasiveness of non-acidic melanoma cells and that a mixed population of acidic and non-acidic cells induced invasive and metastatic potential [105]. The specific role of acidosis derived EVs in invasion and migration was further evaluated, proposing that exosomes secreted by acidic cells (including short-term and long-term, acidosis-adapted cancer cells) induced migration and invasion in recipient cells [78]. We have previously found that hypoxia-derived EVs mimic the hypoxic response in glioma tumor cells, resulting in enhanced *in vivo* tumor growth and remodeling of the stromal cell compartment [106]. Specifically, it was demonstrated that hypoxic tumor cells release pro-coagulant EVs, resulting in pro-tumorigenic effects in recipient stromal cells [107]. Moreover, the proteomic profile of EVs derived from tumor cells cultured in acidic pH (6.0) was altered, showing an increased expression of proteins involved in migration, invasion, metastasis, and survival [78]. Thus, it appears conceivable that acidosis-derived EVs, similarly to EVs from hypoxic conditions, take part in driving disease progression by conditioning of stromal cells as well as local remodeling of the ECM compartment in the primary tumor [108].

Importantly, EVs may also contribute to the generation of a suitable microenvironment at distant sites by conditioning and educating the PMN. The concept of PMN holds that cancer cells in the primary tumor secrete factors that can impact vascular integrity, ECM remodeling, and recruitment and reprogramming of bone marrow–derived cells (BMDCs), favoring future colonization by circulating cancer cells. The general role for EVs in PMN formation has been extensively reviewed elsewhere [109–112]. Here, it should be underlined that TME stress has in several studies been shown to regulate EV cargo composition [106, 113] as well as to promote vesicle shedding [79–81]. Erler and colleagues showed that hypoxia in the primary tumor can induce the secretion of lysyl oxidase (LOX) resulting in collagen cross-linking and ECM remodeling at distant sites that favor recruitment of BMDCs and eventual metastatic seeding [114]. Notably, we found LOX to be highly enriched in hypoxia-derived EVs [106] and others found LOX-like 2 (LOXL2) to be increased on the exterior of EVs derived from hypoxic endothelial cells [115]. Hence, hypoxia-derived EVs may function as an important delivery mechanism of LOX and LOXL2 as well as other actors involved in PMN formation. In addition, metabolic effects of tumor-derived EVs may modulate the metabolic status of cells at distant sites [103] as an additional mechanism whereby EVs take part in PMN conditioning.

## Exploiting the endocytosis machinery for tumor drug delivery—finding the targets

We have discussed several examples of how metastatic cells of the acidic tumor niche fulfill their metabolic demands through induced uptake and utilization of extracellular ligands, such as EVs and lipoproteins. However, similar transport mechanisms may be harnessed for specific delivery of therapeutic substances to the stressed TME. Notably, several vesicle transport mechanisms are deregulated by stress factors of the TME [116]. As a consequence, membrane transport mechanisms have attracted increasing attention as portals of entry for tumor-specific delivery of therapeutic substances, such as antibody–drug conjugates (ADCs). The concept of ADC treatment is to repurpose an antibody as a toxin delivery vehicle to specifically kill tumor cells by intracellular release of the drug. ADCs targeting epidermal growth factor receptor 2 (HER-2) and CD20 have been approved in the treatment of breast cancer and lymphoma, and more than 40 ADCs are currently in clinical trials [117]. However, the identification of internalizing surface tumor antigens of the TME remains an unmet need of high clinical relevance. We have developed a procedure for functional mapping of the cell-surface proteome and recently revealed that caveolin-1 acts as a global negative regulator of receptor internalization in hypoxic cells [118]. As an exception to the general downregulation of receptor internalization by hypoxia, carbonic anhydrase 9 (CA9), among others, was identified as a highly hypoxia specific, cell-surface receptor that could escape from the gate keeper function of caveolin-1. CA9 is highly induced at hypoxic and acidic conditions [119] and constitutes a major player in the regulation of tumor pH homeostasis [120, 121] by the reversible hydration of CO_2_ into HCO_3_^−^ + H^+^. Together with bicarbonate transport proteins, CA9 facilitates intracellular alkalinization with concomitant extracellular proton accumulation and acidification (see Bødkjer, this volume). In normal physiology, CA9 expression is restricted to a few organs including the upper gastrointestinal tract and gallbladder [122]. CA9 thus emerges as an attractive treatment target due to its specific expression in the stressed TME that further associates with worse patient prognosis [119]. Accordingly, several small molecule inhibitors and antibody-based drugs targeted at CA9 are currently explored in experimental models as well as in clinical trials [123] (see Pastorekova, this volume). We recently provided first evidence that CA9 exists in a GAG substituted version, i.e., as a part-time PG. Notably, the GAG-modified version of CA9 (PG-CA9) showed an impaired internalization in cancer cells due to translocation of PG-CA9 to caveolin-1 enriched membrane regions [124]. Based on these and other findings, it may be proposed that perturbation of CA9 glycosylation and/or caveolin-1 provides improved opportunities for enhanced targeting of the acidic tumor niche.

## Conclusions

Deregulated lipid metabolism by the abnormal transfer not only of EVs and lipoproteins through cell-surface PGs but also of other extracellular nutrients and intracellular mechanisms, in the acidic tumor niche results in alteration of vital functions related to stress adaptation and enhanced metastatic potency. It is thus hypothesized that stressed-induced recruitment of lipoproteins and EVs represents a new mechanism of cancer cell adaptation. However, the details of EV/lipoprotein entry and cargo transfer into recipient cells, and how these events are regulated by acidosis, remain open and important questions. Thus, a major goal of future studies should be to gain a better understanding of how receptor complexes cooperate during uptake and intracellular sorting of extracellular lipoprotein particles and EVs. This opens a broad horizon of questions on the interplay between lipoprotein and EV particles in tumor biology: (1) Do they share/compete for similar uptake and signaling pathways in hypoxic/acidic tumor cells? (2) Do they follow similar intracellular sorting routes and metabolic pathways, e.g., as substrates for LD formation? and (3) What is the functional role of lipid loading at the levels of signaling activation, proliferation, migration, and survival *in vitro* as well as tumor cell metastasis *in vivo*? Moreover, further work is necessary to examine how metastatic cells mobilize and utilize lipids stored in LDs, i.e., are LDs consumed during periods of re-oxygenation/pH neutralization to resist cell death by oxidative stress? Could cancer cells support their survival through autophagy-dependent consumption of LDs during their journey in the well-oxygenated blood stream? Finally, how could the craving for extracellular lipids and other nutrient sources in the acidic TME be exploited therapeutically? Together, these avenues for future investigations should provide novel insights at the interface between metabolic disease (obesity) and cancer and establish the increased demand of extracellular lipid particles and lipid storage in stressed cancer cells as a new target for therapeutic intervention of metastasis.
